# LncRNA MSTO2P promotes colorectal cancer progression through epigenetically silencing CDKN1A mediated by EZH2

**DOI:** 10.1186/s12957-022-02567-5

**Published:** 2022-03-26

**Authors:** Mengjun Guo, Xiling Zhang

**Affiliations:** Department of Anus and Intestine Surgery, Shaanxi Provincial People’s Hospital, West Youyi Road, Xi’an, 710000 Shaanxi China

**Keywords:** Pseudogene, MSTO2P, CDKN1A, CRC

## Abstract

**Background:**

Pseudogene-derived long non-coding RNAs (lncRNAs) have been reported to act as key regulatory factors of cancers. However, the study focused on pseudogene misato family member 2 (MSTO2P) in the occurrence and development of colorectal cancer (CRC) remains unclear.

**Methods:**

CCK-8, colony formation, and transwell assays clarified HT-29 and SW480 cell proliferation and invasion. Furthermore, flow cytometry was carried out to detect cell cycle and cell apoptosis. Subcellular localization assay indicated the location of MSTO2P in HT-29 cells. RIP and CHIP assays clarified the relationship of MSTO2P with target protein and gene in HT-29 cells.

**Results:**

MSTO2P expression was upregulated in CRC tissues and cells. Functional experiments revealed that inhibition of MSTO2P suppressed HT-29 and SW480 cell proliferation and invasion, and promoted cell cycle arrest and cell apoptosis. Besides, MSTO2P epigenetically down-regulated cyclin-dependent kinase inhibitor 1A (CDKN1A) via binding to the enhancer of zeste homolog 2 (EZH2) in the nucleus. At last, rescue experiments proved the anti-tumor effect of inhibition of MSTO2P was partially recovered due to the knockdown of CDKN1A in HT-29 cells.

**Conclusion:**

LncRNA MSTO2P promoted colorectal cancer progression through epigenetically silencing CDKN1A mediated by EZH2.

## Background

Colorectal cancer (CRC) is one of the most common malignancies in the digestive tract [1]. According to global cancer statistics in 2020, CRC has the second mortality of cancer death worldwide [2]. The comprehensive treatment methods of CRC are mainly surgery combined with neoadjuvant therapy, postoperative adjuvant therapy, and other therapies [1, 3]. With the understanding of CRC and the continuous improvement of treatment methods, the mortality rate of CRC has been reduced, and the 5-year survival rate has been increased [4]. However, the diagnosis of CRC is still difficult because of the inconspicuous early symptom [5]. Moreover, the recurrence and metastasis rates of CRC are still high after surgery [6]. Therefore, more studies still need to be done to excavate molecules with high diagnostic efficacy for early diagnosis of CRC and further explore the mechanism of its occurrence and prognosis.

Studies have shown that only 1.5–2.0% of genes in the entire genome have the protein-coding capability, and over 90% of sequences are non-coding genes, including pseudogenes, microRNAs (miRNA), and long non-coding RNAs (lncRNAs) [7–9]. Pseudogenes have DNA sequences similar to a functional gene, which lose the original function because of many mutations [7]. Pseudogenes play an important role in various diseases [10, 11]. Some pseudogenes transcribed lncRNAs and were abnormally expressed in cancers [12, 13]. In addition, pseudogene-derived lncRNAs take part in the progression of tumors, acting as proto-oncogenes or tumor suppressor genes [12, 13]. CRC development is a complex and multi-step process, which involves genetic and epigenetic changes to mRNA and non-coding RNAs (ncRNAs) [14, 15]. However, the function of pseudogene-derived lncRNAs in the occurrence and development of CRC remains unclear.

LncRNA misato family member 2 (MSTO2P) is considered a pseudogene and is upregulated in several cancers [16–18]. Furthermore, lncRNA MSTO2P participates in the development of cancers [16–18]. However, studies about whether lncRNA MSTO2P could regulate the CRC process remain limited. The Cancer Genome Atlas (TCGA) database showed that lncRNA MSTO2P was significantly upregulated in CRC tissues, suggesting that it might have important regulatory functions in the tumorigenesis of CRC. The present study aimed to explore the role and molecular mechanism of lncRNA MSTO2P on CRC progression. In the study, lncRNA MSTO2P was upregulated in CRC, and inhibition of MSTO2P suppressed CRC progress. Moreover, MSTO2P epigenetically downregulated cyclin-dependent kinase inhibitor 1A (CDKN1A) via enhancer of zeste homolog 2 (EZH2). Our study provides the first evidence that MSTO2P functions as a potential target for the diagnosis and treatment of CRC.

## Materials and methods

### Tissues samples collection

Fifty-six paired CRC tissues and adjacent normal tissues were obtained from patients at Shaanxi Provincial People’s Hospital between October 2017 and November 2018. The characteristics of all CRC samples were provided in Table 1. All participants did not receive chemotherapy or radiotherapy before tumor removal.

**Table 1:** General clinicopathological characteristics of patients

Characteristics	***N***=56
**Age (years)**	
≤ 60	34 (60.71%)
> 60	22 (39.29%)
**Gender**	
Male	40 (71.43%)
Female	16 (28.57%)
**Diameter of tumor (cm)**	
≤ 5	26 (46.43%)
> 5	30 (53.57%)
**Pathological grading**	
I/II	15 (26.79%)
III/IV	41 (73.21%)
**Distant metastasis**	
Negative	27 (48.21%)
Positive	29 (51.79%)
**TNM stage**	
I/II	33 (58.93%)
III/IV	23 (41.07%)

### Cell culture

NCM460, HT-29, SW480, HCT116, and LOVO cell lines were cultured in DMEM (Gibco, USA) supplemented with 10% FBS (Gibco, USA) in a humidified incubator under 5% CO_2_ at 37°C.

### Cell transfection

To knockdown MSTO2P, CDKN1A, and EZH2, small interfering RNA (siRNA) targeting MSTO2P, CDKN1A, and EZH2 were synthesized by GenePharma (Shanghai, China): SiRNA for MSTO2P (si-MSTO2P), 5′-GGTGGACTCTACAGGGACAAA-3′; siRNA for CDKN1A (si-CDKN1A), 5′-GAAGACCATGTGGACCTGTCA-3′; siRNA for EZH2 (si-EZH2), 5′-GACGAGCTGATGAAGTAAAGA-3′; and siRNA for negative control (si-NC), 5′-UUCUCCGACGUGUCACGUTT-3′. For cell transfection, 50 nM of siRNA was transfected into cells by RNAiMax transfection (Invitrogen, USA) for 48 h following with manufacturer’s instructions.

### RT-qPCR assay

The total RNA was exacted and reversely transcribed to cDNA by the TRIzol and Reverse Transcription Kit (Takara, Japan). Then, cDNA reactions were amplified using SYBR Premix Ex TaqII (Takara, USA) by fluorescent quantitative PCR 7500 (ABI, USA) as recommended by the supplier. For data analysis, U6 and GAPDH were served as the controls. The levels were measured by 2^−ΔΔCT^. The primer sequences were shown as follows: MSTO2P forward, 5′-GTTGCTTTGCCCAGTCAGTG-3′ and MSTO2P reverse, 5′-AACTCATGACTCCAGGCTGC-3′; CDKN2B forward, 5′-GGACTAGTGGAGAAGGTGCG-3′and CDKN2B reverse, 5′-TCATCATGACCTGGATCGCG-3′; CDKN1A forward, 5′-CTGCCCAAGCTCTACCTTCC-3′ and CDKN1A reverse, 5′-TCGACCCTGAGAGTCTCCAG-3′; Bcl-2 forward, 5′-GAACTGGGGGAGGATTGTGG-3′ and Bcl-2 reverse, 5′-CATCCCAGCCTCCGTTATCC-3′; Bax forward, 5′-AAGGTGCCGGAACTGATCAG-3′ and Bax reverse, 5′-GTCTTGGATCCAGCCCAACA-3′; EMP1 forward, 5′-CGCAGTATCACCACGGCTAT-3′ and EMP1 reverse, 5′-GGACCAGATAGAGAACGCCG-3′; PTEN forward, 5′-TGGTCTGCCAGCTAAAGGTG-3′ and PTEN reverse, 5′-ACACACAGGTAACGGCTGAG-3′; GAPDH forward, 5′-GATTTGGTCGTATTGGGCGC-3′ and GAPDH reverse, 5′-TTCCCGTTCTCAGCCTTGAC-3′; U6 forward, 5′-GCTTCGGCAGCACATATACTAA-3′ and U6 reverse, 5′-AACGCTTCACGAATTTGCGT-3′.

### Cell counting kit-8 (CCK-8) assay

The viability of HT-29 and SW480 cells was analyzed by CCK-8 assay (Beyotime, China). In Brief, cells were seeded into 96-well plates for 24 h, 48 h, and 72 h. Afterward, 10 μL of CCK-8 solution was incubated with cells at 37 °C for 2 h. The absorbance at 450 nm was detected by a microplate reader (Bio-Rad, USA).

### Colony formation assay

Cells were inoculated into 6-well plates at a density of 5×10^3^ cells/well for 14 days. Then, cells were fixed with methanol (Solarbio, China) for 20 min and stained with 0.5% crystal violet (Solarbio, China) for 15 min. The cell colonies were photographed with a light microscope (Olympus, Japan) and counted by a naked eye.

### Invasive transwell assay

The invasion ability of HT-29 and SW480 cells was detected by a transwell assay. Cells with the serum-free medium were seeded to the upper transwell chamber (Costar, USA) with precoated Matrigel (BD biosciences, USA). The bottom of the chamber was supplied with DMEM containing 10% FBS. After 24 h, the invaded cells were fixed in 4% paraformaldehyde (Solarbio, China) for 20 min and stained with 0.5% crystal violet (Solarbio, China) for 15 min. The invaded cells were imaged with a light microscope (Olympus, Japan), and the cell number was calculated.

### Flow cytometry assay

Flow cytometry assay was performed to investigate the cell cycle and apoptosis. HT-29 and SW480 cells were collected by trypsin (Gibco, USA). For cell cycle detection, cells were fixed in 75% ethanol (Beyotime, China) overnight and PI (Beyotime, China) for 15 min in the dark. For cell apoptosis detection, cells were incubated with Annexin V-FITC (Beyotime, China) and PI (Beyotime, China) for 15 min in the dark. Subsequently, the cell cycle and apoptotic cells were detected by a FACScalibur Flow Cytometry (BD Biosciences, USA).

### Subcellular fractionation location

RNA Subcellular Isolation Kit (Active Motif, USA) was used to segregate and purify the cytoplasmic and nuclear RNA from HT-29 cells. Then, the collected RNA was transcribed to cDNA and analyzed by RT-qPCR assay. For data analysis, GAPDH was used as the cytoplasmic control, and U6 was used as the nuclear control.

### RNA immunoprecipitation (RIP) assay

RIP assay was used to explore the interaction between MSTO2P and EZH2 in HT-29 cells using Imprint RIP Kit (Millipore, USA). In brief, cells were lysed in a complete lysis buffer for 30 min at 4 °C. Then, the cell supernatant was incubated with magnetic beads bound with IgG antibody (Abcam, USA) or EZH2 antibody (Abcam, USA) for 4 h at 4 °C. After incubation, the beads were washed and eluted. Then, proteinase K was added to remove protein at 55 °C for 30 min. The input and immunoprecipitated RNAs were isolated to assess MSTO2P expression using RT-qPCR assay.

### Chromatin immunoprecipitation (CHIP)-qPCR assay

ChIP assay was used to evaluate whether MSTO2P recruited EZH2 to CDKN1A promoter region and resulted in trimethylation of H3K27 in HT-29 cells. ChIP assay was carried out by EZ-ChIP kit (Millipore, USA). In brief, HT-29 cells with si-MSTO2 transfection were incubated with formaldehyde to produce cross-linked chromatin. Then, the cross-linked chromatin was sonicated to 200 to 300 bp chromatin fragments. Equal amounts of fragments were immunoprecipitated with anti-lgG (Abcam, USA), anti-EZH2 (Abcam, USA), or anti-H3K27me3 (Abcam, USA) overnight. After incubation, the precipitated chromatin DNA was isolated to assess CDKN1A expression using RT-qPCR assay. The primer sequences of CDKN1A for CHIP-qPCR were shown as follows: forward, 5′-GCCTTCCTCACATCCTCC-3′ and reverse, 5′-CAAGAGTGCCCAGTCCAG-3′.

### Western blot

The total protein was isolated from HT-29 cells using $\mathrm{RIPA}\ \mathrm{lysis}\ \mathrm{buffer}\ \mathrm{plus}\ \mathrm{PMSF}\ \left(\mathrm{Beyotime},\mathrm{China}\right).$The total protein concentration was detected by a BCA kit (Beyotime, China). Then, 20 μg protein samples were separated by SDS-PAGE gels and transferred into the PVDF membranes. Specific primary antibodies anti-CDKN1A (ab102013, 1:1000, Abcam, USA) and anti-GAPDH (ab8245, 1:5000, Abcam, USA) were incubated at 4 °C overnight. Afterward, the membranes were probed with a secondary antibody for 2 h. At last, the images were collected by ECL luminescence (Beyotime, China).

### Statistical analysis

The data were displayed as the mean ± SEM of 3 independent experiments. The statistical analysis was performed using the Prism Graphpad 8.0 software. Two-tailed Student’s *t* test was used to analyze differences between two groups. One-way analysis of variance (ANOVA) followed by Tukey’s test was used to analyze differences among multiple groups. P<0.05 was considered to indicate a statistically significant difference.

## Results

### LncRNA MSTO2P is aberrantly overexpressed in CRC tissues and cells

**Fig. 1:**
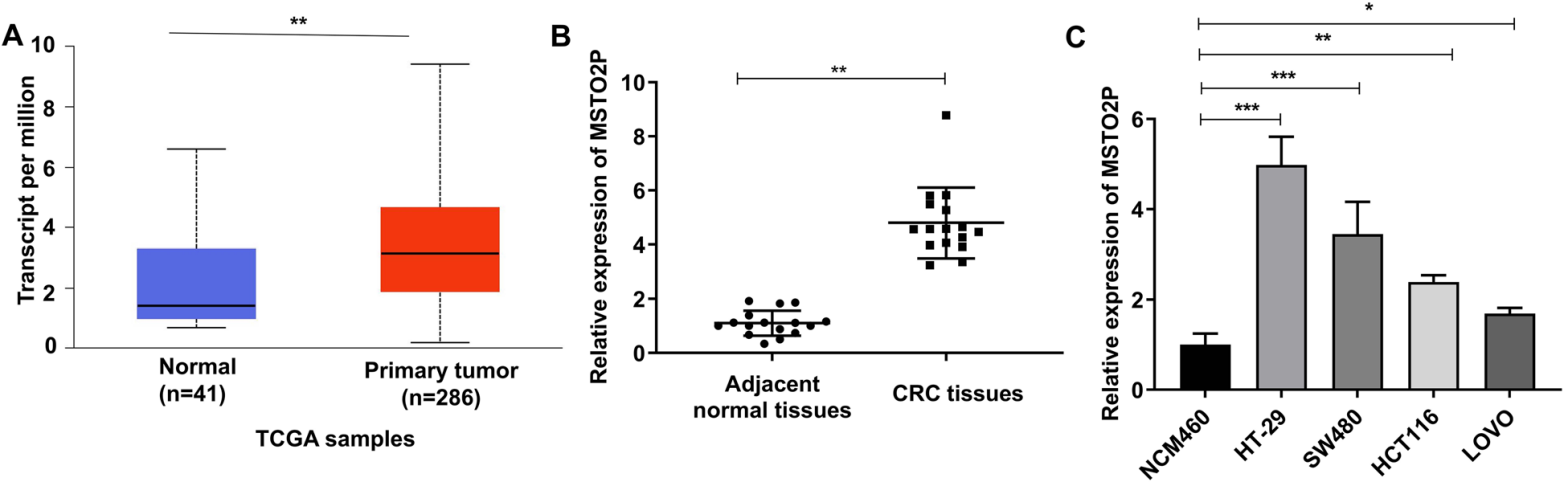
LncRNA MSTO2P is aberrantly overexpressed in CRC tissues and cells. **A** TCGA database showed that MSTO2P level was increased in primary CRC tissues. **B** RT-qPCR assay revealed MSTO2P level was markedly upregulated in CRC tissues. **C** RT-qPCR assay indicated high level of MSTO2P in CRC cell lines. *p<0.05, **p<0.01, and ***p<0.001

MSTO2P level was identified in CRC tissues using TCGA database and RT-qPCR assay. TCGA database showed that the MSTO2P level was increased by 2.13 fold in CRC tissues compared with colon tissues (Fig. 1A). In addition, the RT-qPCR assay suggested that the MSTO2P level was markedly higher in 56 of CRC tissues than in the adjacent normal tissues (Fig. 1B). Meanwhile, increased MSTO2P was also observed in CRC cell lines (HT-29, SW480, HCT116, LOVO) compared with normal epithelial cell lines (NCM460) (Fig. 1C). As HT-29 and SW480 cells exhibited the highest level of MSTO2P (3.98-fold and 2.45-fold, respectively), HT-29 and SW480 cells were chosen for the next experiments. Above all, these data showed MSTO2P level was highly expressed in CRC.

### Inhibition of MSTO2P suppresses proliferation and invasion of CRC cells

**Fig. 2:**
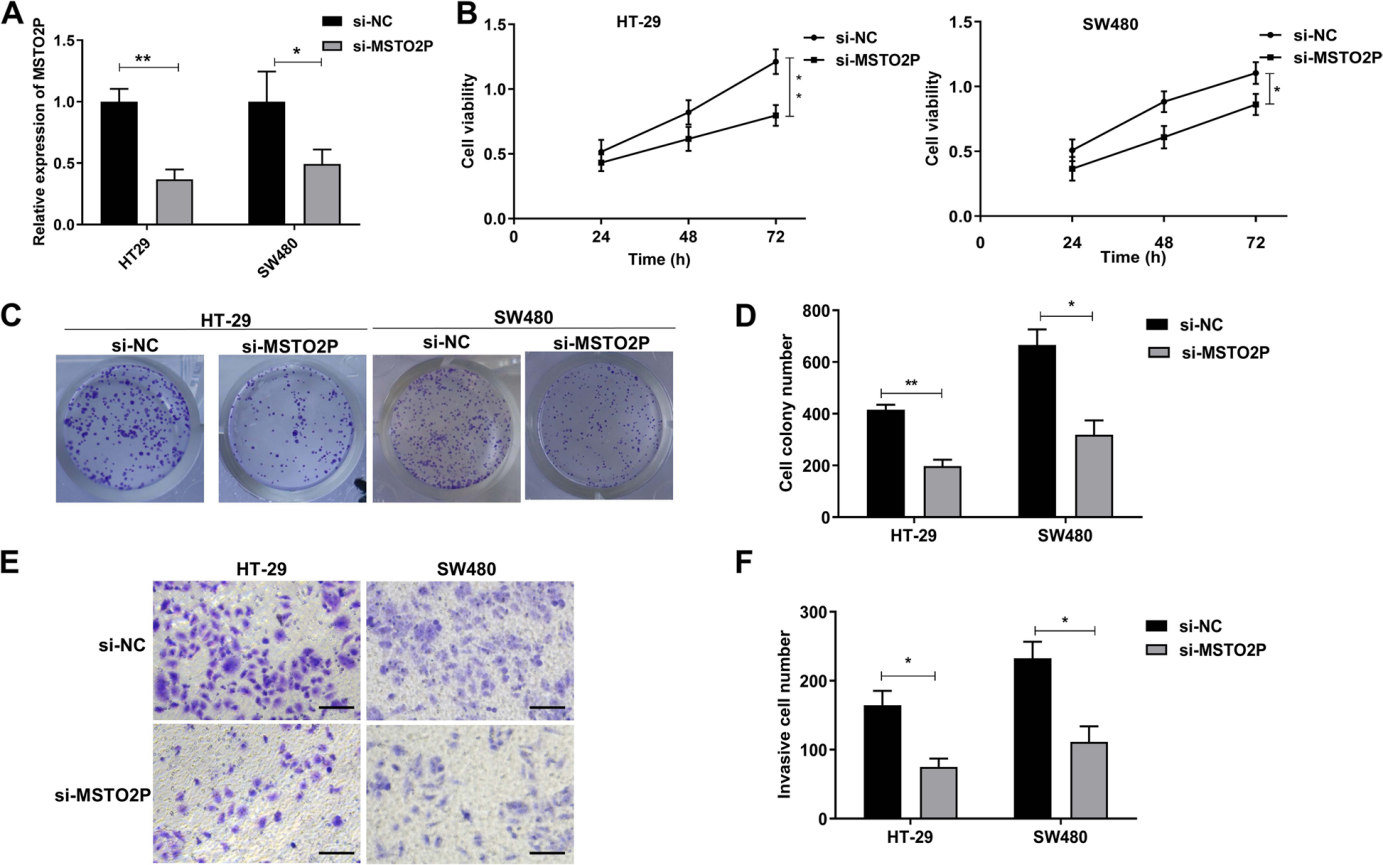
Inhibition of MSTO2P suppresses proliferation and invasion of CRC cells. MSTO2P was knocked down in HT-29 and SW480 cells by transfecting cells with si-MSTO2P for 24 h. **A** The transfection efficiency of si-MSTO2P was confirmed by RT-qPCR assay. **B** CCK-8 assay demonstrated that knockdown of MSTO2P dramatically suppressed cell viability. **C**, **D** Colony formation assay clarified that suppression of MSTO2P reduced cell colonies. **E**, **F** Transwell assay displayed that the downregulation of MSTO2P significantly suppressed cell invasion. Bar values= 100 μm. *p<0.05, **p<0.01, and ***p<0.001

To illuminate the roles of MSTO2P in CRC, si-NC and si-MSTO2P were transfected into HT-29 and SW480 cells, respectively. The transfection efficiency of si-MSTO2P was confirmed by RT-qPCR assay (Fig. 2A). CCK-8 assay demonstrated that knockdown of MSTO2P dramatically suppressed HT-29 and SW480 cell viability (0.53-fold and 0.28-fold, respectively) (Fig. 2B). Furthermore, colony formation assay clarified that suppression of MSTO2P reduced the number of colonies in HT-29 and SW480 cells (1.11-fold and 1.09-fold, respectively) (Fig. 2C, D). Subsequently, the transwell assay displayed that the downregulation of MSTO2P significantly decreased the invasion of HT-29 and SW480 cells (1.18-fold and 1.09-fold, respectively) (Fig. 2E–F). Overall, our findings elucidated that downregulation of MSTO2P inhibited cell proliferation and invasion in CRC.

### Inhibition of MSTO2P promotes cell cycle arrest and cell apoptosis

**Fig. 3:**
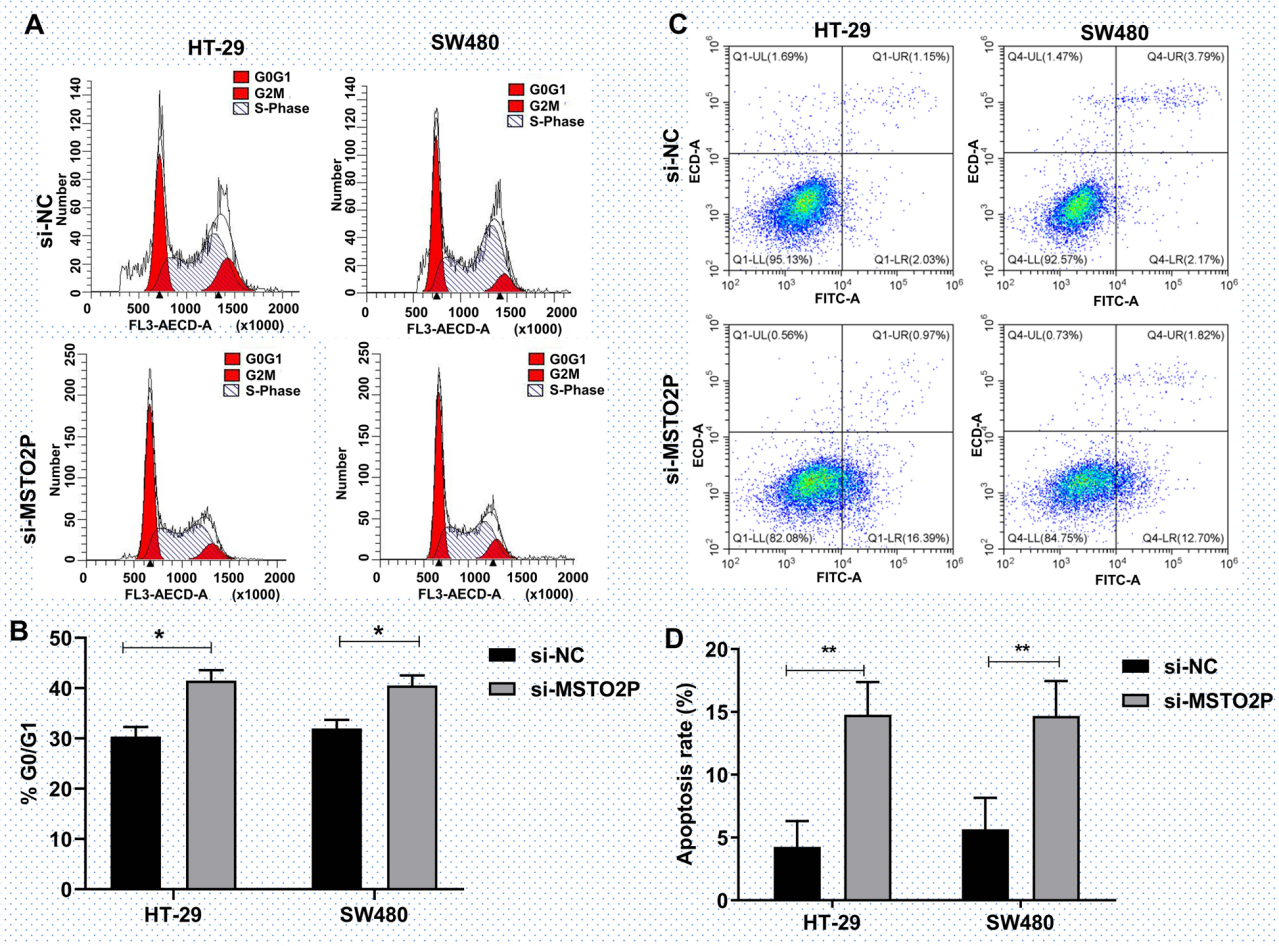
Inhibition of MSTO2P promotes cell cycle arrest and cell apoptosis. **A**, **B** Flow cytometry assay showed that the proportion of HT-29 and SW480 cells at the G0/G1 phase was elevated by si-MSTO2P. **C**, **D** Flow cytometry assay demonstrated that si-MSTO2P enhanced cell apoptosis. *p<0.05, **p<0.01, and ***p<0.001

We then investigated the effect of MSTO2P on the cell cycle and cell apoptosis. The result showed that the proportion of HT-29 and SW480 cells at the G0/G1 phase was significantly elevated by the downregulation of MSTO2P (Fig. 3A, B). Moreover, knockdown of MSTO2P enhanced HT-29 and SW480 cell apoptosis (Fig. 3C, D). Collectively, these findings demonstrated that MSTO2P participated in CRC progression through regulating the cell cycle and apoptosis.

### MSTO2P interacts with EZH2 to inhibit CDKN1A transcription

**Fig. 4:**
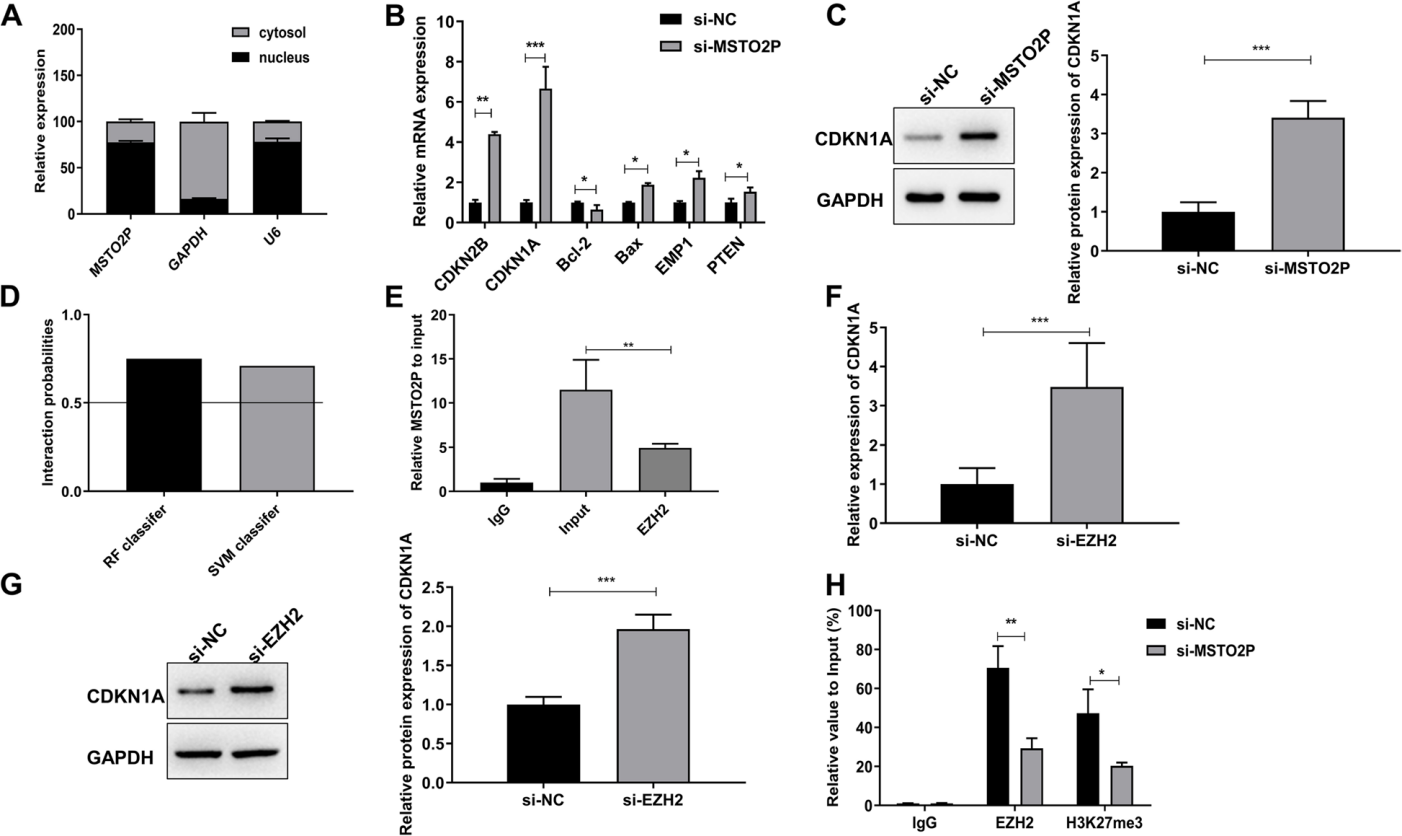
MSTO2P interacts with EZH2 to inhibit CDKN1A transcription. **A** The distribution of MSTO2P in HT-29 cells was evidenced by RT-qPCR assay. **B** RT-qPCR assay showed that si-MSTO2P upregulated CDKN2B, CDKN1A, Bax, EMP1, and PTEN expression level and decreased Bcl-2 level in HT-29 cells. **C** Western blot assay showed that si-MSTO2P upregulated CDKN1A protein level. **D** Bioinformatics were used to predict the interaction probabilities of lncRNA MSTO2P and EZH2 via RNA-protein interaction prediction (http://pridb.gdcb.iastate.edu/rpiseq/). Predictions with probabilities >0.5 were considered positive. **E** RIP assay confirmed that MSTO2P was directly bound to EZH2 in HT-29 cells. **F** RT-qPCR assay showed si-EZH2 significantly enhanced CDKN1A expression level. **G** Western blot assay showed si-EZH2 significantly enhanced CDKN1A expression level. **H** CHIP assay showed that si-MSTO2P suppressed binding of EZH2 and H3K27me3. *p<0.05, **p<0.01, and ***p<0.001

The distribution of MSTO2P in HT-29 cells was evidenced by a subcellular localization assay. We found that MSTO2P was markedly located in the nucleus (Fig. 4A). It is reported that lncRNAs participate in various disease processes via interacting with specific RNA-binding proteins [19]. We then wondered whether MSTO2P regulated CRC progression through cell proliferation, cycle, apoptosis-related transcripts. The RT-qPCR assay showed that si-MSTO2P upregulated CDKN2B, CDKN1A, Bax, EMP1, and PTEN expression level and decreased Bcl-2 level in HT-29 cells (Fig. 4B). Importantly, CDKN1A had the largest fold difference in cells with si-MSTO2P transfection. Western blot assay also showed that si-MSTO2P upregulated CDKN1A protein level (Fig. 4C). Furthermore, the online database RPISeq (http://pridb.gdcb.iastate.edu/RPISeq/) was used to predict the potential chromatin modifiers of CDKN1A. According to bioinformatics analysis, EZH2 (H3K27me3) might be the binding protein of MSTO2P (as the support vector machine [SVM] or random forest [RF] score >0.5; Fig. 4D). RIP assay confirmed that MSTO2P was directly bound to EZH2 in HT-29 cells (Fig. 4E). In addition, the RT-qPCR assay showed that knockdown of EZH2 significantly enhanced CDKN1A expression level (Fig. 4F). Western blot assay also showed that knockdown of EZH2 significantly enhanced CDKN1A protein level (Fig. 4G). CHIP assay showed that inhibition of MSTO2P suppressed binding of EZH2 and H3K27me3 across the CDKN1A promoter region (Fig. 4H). These data provided strong evidence that MSTO2P acted as a scaffold through interacting with EZH2, thus epigenetically inhibiting CDKN1A in the nucleus.

### Inhibition of MSTO2P suppresses CRC progression through up-regulating CDKN1A expression

**Fig. 5:**
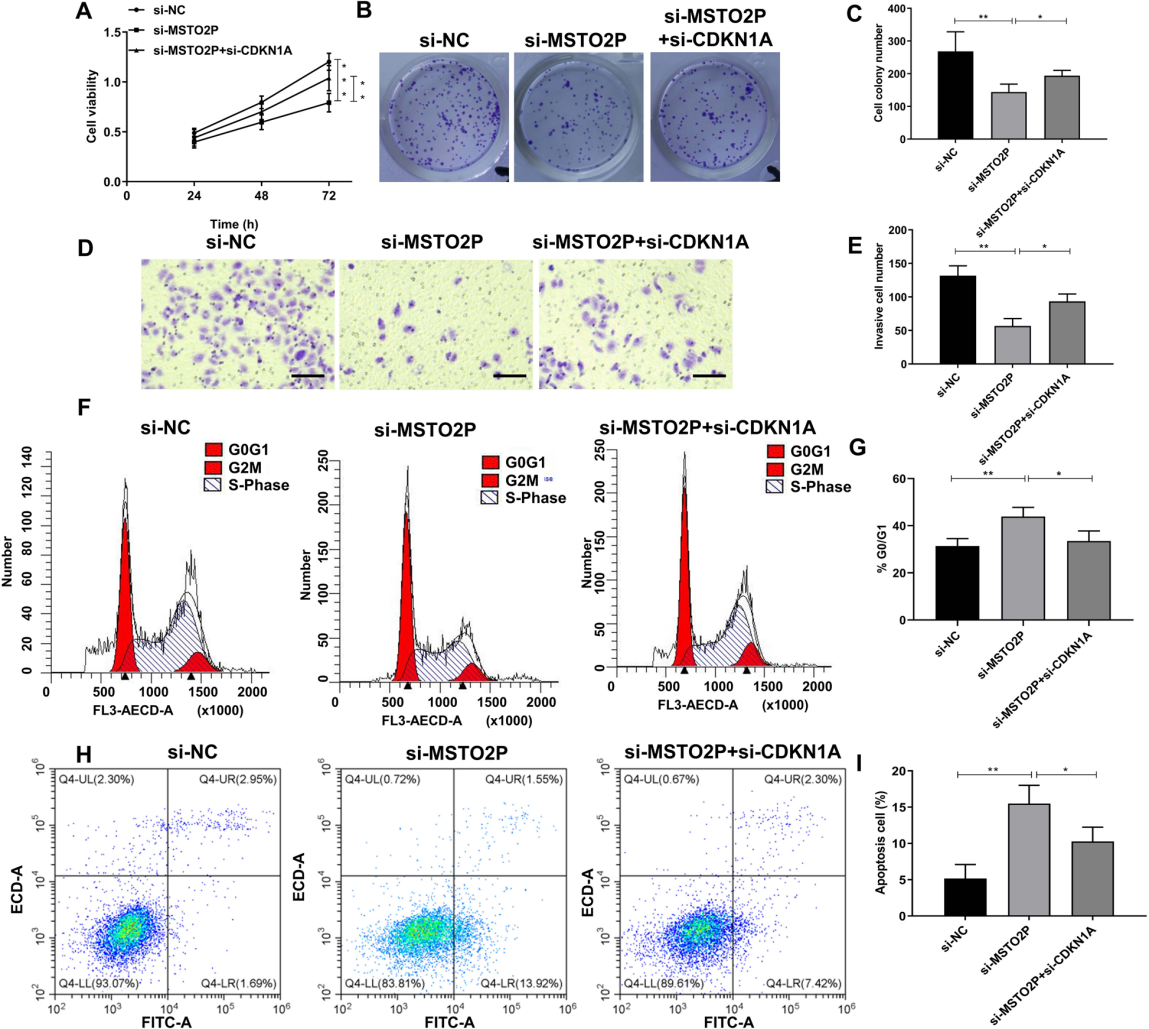
Inhibition of MSTO2P suppresses CRC progression through upregulating CDKN1A expression. si-MSTO2P and si-CDKN1A were co-transfected into HT-29 cells. **A** CCK-8 assay showed that cell viability was inhibited by si-MSTO2P, which was reversed by si-CDKN1A. **B**, **C** Colony formation assay clarified that si-MSTO2P decreased the number of colonies in HT-29, while si-CDKN1A partially recovered clone formation. **D**, **E** Transwell assay showed cell invasion in HT-29 cells. Bar values= 100 μm. **F**, **G** The cell cycle and (**H**–**I**) cell apoptosis were detected by flow cytometry assay

To confirm whether MSTO2P executed its function in CRC through regulating CDKN1A, HT-29 cells were co-transfected with si-MSTO2P and si-CDKN1A. The results demonstrated the decreased proliferation and invasion of HT-29 cells caused by inhibition of MSTO2P were partially recovered due to knockdown of CDKN1A (Fig. 5A–E). Furthermore, inhibition of MSTO2P-induced cell cycle arrest and apoptosis, while knockdown of CDKN1A reversed the effect of inhibition of MSTO2P (Fig. 5F–I). Overall, these data suggested that MSTO2P participated in CRC cell malignancy through regulating CDKN1A.

## Discussion

Emerging studies have emphasized the significance of pseudogenes and revealed the importance of pseudogene-derived lncRNAs in the occurrence and development of tumors [20, 21]. Previous studies revealed that pseudogene DUXAP8 fascinated CRC cell proliferation and invasion [22, 23]. Besides, pseudogene TPTE2P1 was increased in CRC and promoted CRC progression and aggressiveness [24]. It was reported pseudogene MSTO2P played an oncogenic role via targeting miR-335 level in gastric cancer [16]. In addition, Wang et al. suggested MSTO2P accelerated lung cancer cell proliferation and autophagy [17]. Furthermore, MSTO2P was proved to promote osteosarcoma progression under hypoxia through increasing PD-L1 level [25].

In the present study, the MSTO2P level was highly increased in CRC. Considering the carcinogenic effect of MSTO2P in previous studies, we speculated that MSTO2P might exert as an oncogene to control CRC progression. To clarify the biological role of MSTO2P in CRC, we performed loss-of-function experiments in HT-29 and SW480 cells. We found that inhibition of MSTO2P suppressed the malignancy of CRC, as indicated by which decreased CRC cell proliferation, invasion, and promoted cell cycle arrest and apoptosis.

The role of pseudogene-derived lncRNAs in cells mainly depends on the interactions with other biomolecules [26, 27]. On the one hand, lncRNAs located in the cytoplasm participate in the post-transcriptional regulation by interacting with other proteins or RNA molecules [27]. For instance, DUXAP8 was mainly expressed in the cytoplasm and played the oncogenic role by sponging miR-577 in CRC [23]. On the other hand, lncRNAs located in the nucleus can bind with other proteins or transcription factors in the form of RNA to participate in the transcription regulation of genes [28]. For instance, Lian et al. suggested that DUXAP8 promoted metastasis of pancreatic carcinoma by suppressing CDKN1A and KLF2 levels by interacting with EZH2 and LSD1 in the nucleus [29].

To further explore the molecular mechanisms that DUXAP8 might be involved in CRC, we performed a subcellular localization assay. We found that MSTO2P was markedly located in the nucleus, indicating that MSTO2P might exert biological roles at the transcriptional level. To search the target gene of MSTO2P, we detected the expression of cell proliferation, cycle, apoptosis-related transcripts, such as CDKN2B, CDKN1A, Bax, Bcl-2, EMP1, and PTEN [29–32]. The result suggested that CDKN1A had the largest fold difference in cells with si-MSTO2P transfection. Therefore, we chose CDKN1A for further study. EZH2 is a histone methyltransferase, which is the catalytic subunit of the PRC2 complex [32]. EZH2-mediated histone H3K27me3 in the nucleus plays an important role in epigenetic gene silencing [33]. Evidence has suggested that pseudogene-derived lncRNAs interact with EZH2 in the nucleus and suppress the level of H3K27me3 on genes related to cell proliferation and cycle, thus playing a role in tumors [22, 34]. Additionally, we found that MSTO2P acted as a scaffold through interacting with EZH2, thus epigenetically inhibiting CDKN1A in the nucleus. Besides, knockdown of CDKN1A abolished the anti-tumor effect of inhibition of MSTO2P in HT-29 cells.

It’s reported that aberrant expression of lncRNA is closely associated with various diseases [12, 13]. In the present study, the MSTO2P level was highly increased in CRC. Therefore, MSTO2P could be used as a potential biomarker for the early diagnosis of CRC, as well as prognosis. In addition, MSTO2P promoted CRC progression. Therefore, the exploration of antisense oligonucleotide targeting MSTO2P provided a promising therapeutic strategy for CRC. However, further experiments are necessary before the clinical application due to the off-target event or unstable efficiency.

## Conclusions

In summary, we found that MSTO2P was upregulated in CRC and promoted colorectal cancer progression through epigenetically silencing CDKN1A mediated by EZH2. We identified that MSTO2P was a tumor suppressor gene in CRC, which might provide a new idea for targeted therapy of CRC.

## Data Availability

The datasets used and/or analyzed during the current study are available from the corresponding author on reasonable request.
